# Enhanced Fluorine-19 MRI Sensitivity using a Cryogenic Radiofrequency Probe: Technical Developments and *Ex Vivo* Demonstration in a Mouse Model of Neuroinflammation

**DOI:** 10.1038/s41598-017-09622-2

**Published:** 2017-08-29

**Authors:** Sonia Waiczies, Jason M. Millward, Ludger Starke, Paula Ramos Delgado, Till Huelnhagen, Christian Prinz, Daniel Marek, Didier Wecker, Ralph Wissmann, Stefan P. Koch, Philipp Boehm-Sturm, Helmar Waiczies, Thoralf Niendorf, Andreas Pohlmann

**Affiliations:** 1 Berlin Ultrahigh Field Facility (B.U.F.F.), Max Delbrück Center for Molecular Medicine in the Helmholtz Association, Berlin, Germany; 2 Bruker BioSpin AG, Fällanden, Switzerland; 3 Bruker BioSpin MRI, Ettlingen, Germany; 4 Department of Experimental Neurology, Center for Stroke Research Berlin (CSB), Charité Core Facility 7T Experimental MRIs, and NeuroCure, Charité University Medicine Berlin, Berlin, Germany; 5 MRI TOOLS GmbH, Berlin, Germany; 6 Experimental and Clinical Research Center, a joint cooperation between the Charité Medical Faculty and the Max Delbrück Center for Molecular Medicine in the Helmholtz Association, Berlin, Germany

## Abstract

Neuroinflammation can be monitored using fluorine-19 (^19^F)-containing nanoparticles and ^19^F MRI. Previously we studied neuroinflammation in experimental autoimmune encephalomyelitis (EAE) using room temperature (RT) ^19^F radiofrequency (RF) coils and low spatial resolution ^19^F MRI to overcome constraints in signal-to-noise ratio (SNR). This yielded an approximate localization of inflammatory lesions. Here we used a new ^19^F transceive cryogenic quadrature RF probe (^*19*^ *F*-*CRP*) that provides the SNR necessary to acquire superior spatially-resolved ^19^F MRI. First we characterized the signal-transmission profile of the ^*19*^ *F*-*CRP*. The ^*19*^ *F*-*CRP* was then benchmarked against a RT ^19^F/^1^H RF coil. For SNR comparison we used reference compounds including ^19^F-nanoparticles and *ex vivo* brains from EAE mice administered with ^19^F-nanoparticles. The transmit/receive profile of the ^*19*^ *F*-*CRP* diminished with increasing distance from the surface. This was counterbalanced by a substantial SNR gain compared to the RT coil. Intraparenchymal inflammation in the *ex vivo* EAE brains was more sharply defined when using 150 μm isotropic resolution with the ^*19*^ *F*-*CRP*, and reflected the known distribution of EAE histopathology. At this spatial resolution, most ^19^F signals were undetectable using the RT coil. The ^*19*^ *F*-*CRP* is a valuable tool that will allow us to study neuroinflammation with greater detail in future *in vivo* studies.

## Introduction

Central nervous system (CNS) inflammation, as occurs in multiple sclerosis (MS), involves immune cell recruitment from the periphery into the CNS, resulting in tissue destruction and neurodegeneration^1^. During active disease, a massive infiltration of immune cells is predominant, particularly around white matter lesions. T cells find their way into the white matter via a disruption of the blood brain barrier^2^. In MS, T cells may also enter the CNS grey matter such as the cerebral cortex via the meninges^3, 4^. Even in the cerebellum, extensive grey matter pathology in secondary progressive MS is linked to inflammation of the subarachnoid space^5^. Studies of the animal model of MS, experimental autoimmune encephalomyelitis (EAE), have helped identify mechanisms of cell migration between the periphery, CNS and lymphatic system during neuroinflammation^6–8^. This is a topic of active interest, with divergent views regarding immune cell entry and exit in the CNS (inside-out versus outside-in hypotheses) in MS^9, 10^. Therefore there is an acute need for more precise and non-invasive methods that support longitudinal studies of inflammatory cell migration during disease progression to resolve some of the discrepancies in the literature.

Previously we studied immune cell infiltration in EAE brains using fluorine-19 (^19^F)-loaded nanoparticles (NPs) and a room temperature (RT) dual-tuned ^19^F/^1^H radio frequency (RF) volume resonator^11^. Intravenously administered NPs are taken up by inflammatory cells during their migration from the systemic circulation into the inflamed organ^11–17^. Although tracking of inflammation following intravenous ^19^F-NP administration is one application for ^19^F MRI, several other state-of-the-art applications for ^19^F imaging exist. These include *in vivo* tracking of cell therapies labeled in culture with ^19^F-NPs prior to their adoptive transfer^18–20^ and intracellular oximetry using ^19^F-NP emulsions^21^ to study changes in pO2 in tumor cells during therapy^22^.

One major limitation of ^19^F MRI is the low signal-to-noise ratio (SNR). The acquisition method is one aspect of ^19^F MRI that influences SNR. SNR efficiency of the most commonly used acquisition methods — RARE (Rapid Acquisition with Relaxation Enhancement), UTE (Ultra-short Echo Time), and bSSFP (Balanced Steady-State Free Precession) — depends on the *T*_1_ and *T*_2_ values of the particular ^19^F compound studied^23^. For most *T*_1_ and *T*_2_ combinations, especially those pertaining to intracellular ^19^F-NPs, bSSFP and 3D RARE sequences have the highest SNR sensitivity. However, while bSSFP often has a higher SNR efficiency, it is not always the method of choice due to the high RF energy deposition associated with longer acquisition times, and pronounced banding artifacts. The SNR and the sensitivity of the radio frequency (RF) probe used are main determinants that dictate the level of spatial resolution. Factors to be kept in mind when designing a probe include the geometry, the filling factor and the homogeneity of the *B*_1_^+^ transmit field.

The SNR constraint limited spatial resolution to approximately 600 μm when detailing the dynamics of inflammation during EAE^11^. Given this limited precision, the location of inflammatory cells within the brain was not sharply defined. To overcome the sensitivity constraints in ^19^F MR and improve detail of inflammatory cell location, we applied the concept of cryogenically-cooling RF coil hardware to improve SNR by reducing thermal noise. Until now this technology has been available only for ^1^H, ^13^C and ^31^P small animal MRI. Here we made use of the first ^19^F transceive cryogenically-cooled RF probe (^*19*^ *F*-*CRP*) to substantially boost SNR beyond that of available RT coils, thus facilitating the acquisition of better spatially-resolved images. In this study we evaluated the advantages and disadvantages of the ^*19*^ *F*-*CRP* for imaging neuroinflammation.

## Methods

### Radio frequency coils

**Figure 1:**
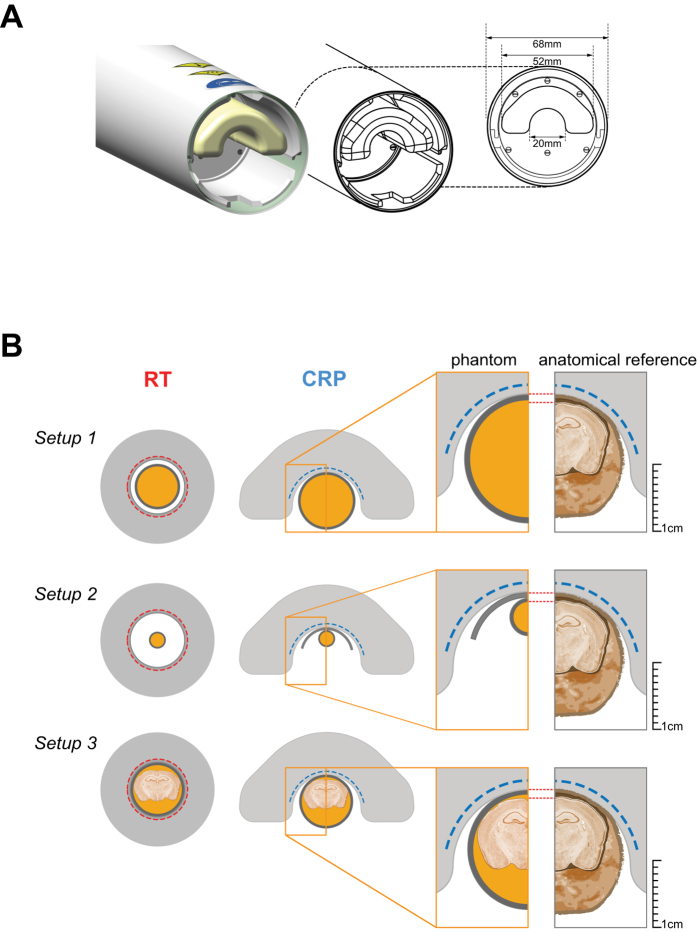
^19^F Cryogenic Radiofrequency Probe design and experimental setup. (**A**) Side view of the ^*19*^ *F*-*CRP* showing its geometry including external protective cylinder and an inner ceramic probe head that encloses the loop coil elements (not shown). The inner diameter dimension for the inner ceramic structure is shown in the cross-sectional view (right). (**B**) Three different experimental setups that were used to assess the ^*19*^ *F*-*CRP* quality. Shown are Setup 1 for the high concentration ^19^F phantom (*upper panel*), Setup 2 for the ^19^F nanoparticle phantoms (*middle panel*) and Setup 3 for the mouse brain phantom (*lower panel*). The dimension of the phantom setups are to scale with the dimensions of both ^*19*^ *F*-*CRP* and RT coil and an anatomic reference is shown on the right for comparison. The nanoparticles used in this study had the following physical characteristics: Z-average diameter = 164 nm, PdI = 0.06, z-Potential = 0.19 mV.

The performance of a novel transceive ^19^F cryogenic quadrature RF surface probe at 9.4T (^*19*^ *F*-*CRP*, *f* ~ 376 MHz) was compared to a dual-tunable ^19^F/^1^H volume resonator (ϕ_in_ = 18.4 mm, l_total_ = 39 mm), previously developed for imaging mouse brain inflammation^11^. The ^*19*^ *F*-*CRP* has a similar geometry to the existing Bruker ^1^H quadrature CryoProbes^24^. The rectangular transceive copper coil elements are overlapping side-by-side on a cylindrical surface (r ~ 11 mm, axis parallel to the main magnetic field direction). The outer dimensions (O.D.) of one coil element are: 16 × 20 mm^2^ [arc length (ϕ × z)] and the total O.D. are: 27 × 20 mm^2^ [ϕ × z]. The ^*19*^ *F*-*CRP* operates at ~28 K with a dual cooled preamplifier at the base running at ~77 K. Constant cooling is ensured by a closed loop system connected to a remote cryo-cooler. The RF coil is thermally insulated by a vacuum separating it from the surrounding ceramic finger (Fig. 1A). The outer surface of the RF finger is equipped with a temperature sensor and kept at a temperature of choice (35 °C) using a resistive heater. The SNR gain of this CRP relative to a RT coil with similar geometry is expected to be comparable to existing 400 MHz proton CryoProbes^24, 25^.

### Experimental setup

To evaluate the ^*19*^ *F*-*CRP* performance, three different phantom-setups were prepared (Fig. 1B):

**Setup 1** (high concentration ^19^F): a 10 ml syringe (inner/outer diameter = 17.0 mm/15.5 mm) for the ^*19*^ *F*-*CRP* and a 5 ml syringe (I.D./O.D. = 13.5/12.0 mm) for the ^*19*^ *F*/^*1*^ *H RT*-*coil*, both containing the same ^19^F reference compound to study ${{\boldsymbol{B}}}_{1}^{+}$ and compare spatial SNR. The reference compound was 33% v/v 2,2,2-Trifluoroethanol (TFE, Sigma-Aldrich, Germany) in water.

**Setup 2** (^19^F nanoparticles): NMR tubes (I.D./O.D. = 4.0/5.0 mm) containing different concentrations of perfluoro-15-crown-5-ether (PFCE) loaded nanoparticles to compare ^19^F signal sensitivity as a function of the number of ^19^F atoms. Nanoparticles were prepared by emulsifying 1200 mM PFCE (Fluorochem, UK) with Pluronic F-68 (Sigma-Aldrich, Germany) using a titanium sonotrode (Sonopuls GM70, Bandelin, Germany) as previously described^26^. The PFCE nanoparticle stock was then diluted to 25 mM, 50 mM, 100 mM, 200 mM, 400 mM and 600 mM nanoparticle suspensions. NMR tubes containing different nanoparticle concentrations were placed below the CRP using a spacer of 0.75 mm thickness to mimic the distance of the mouse brain from the CRP surface in *in vivo* applications.

**Setup 3** (mouse brain): *Ex vivo* tissues from fixed EAE mice embedded in 15-ml tubes, for comparing ^19^F signal sensitivity and anatomical detail. All experiments were conducted in accordance with procedures approved by the Animal Welfare Department of the State Office of Health and Social Affairs Berlin (LAGeSo), and conformed to national and international guidelines to minimize discomfort to animals (86/609/EEC). EAE was induced as described previously^11^ in SJL/J mice (n = 6, female, 6–8 weeks old). Five days following EAE induction, mice were administered nanoparticles (10µmol PFCE) intravenously each day for 5 d as described previously^11^. EAE mice were transcardially perfused with 20 ml PBS followed by 20 ml 4% paraformaldehyde (PFA) following terminal anesthesia. Mice were cleared from external pelt, extremities, and abdominal tissues. Brain, spinal cord and neck lymphoid organs were preserved *in situ* within the skull and vertebral column. The tissues were transferred into a 15 ml tube filled with 4% PFA and stored at 4 °C.

### MRI Methods and Data Analysis

All experiments were carried out on a 9.4 T small animal MR system (BioSpec 94/20, Bruker BioSpin MRI, Ettlingen, Germany) operating at 400 MHz (^1^H) and 376 MHz (^19^F).

#### Transmit Field Characteristics

Using a 15 ml tube containing 33% TFE in water (*Setup 1*), we acquired 2D-FLASH images (TR = 20 s, TE = 4.9 ms, FOV = (20 × 20) mm^2^, matrix = 256 × 256, 1 slice of 4 mm thickness, averages = 1, TA = 1 h 25 min) with nominal excitation flip angles α = 60° and 2α = 120° and calculated the actual flip angles (FA) using the double-angle method^27, 28^:

1 $${\rm{FA}}={\rm{acos}}({{\rm{SI}}}_{2{\rm{\alpha }}}/(2\,{{\rm{SI}}}_{{\rm{\alpha }}}))$$

with SI_α_ and SI_2α_ being the signal intensities obtained with α and 2α. FA maps were normalized to a nominal angle of 90° by multiplying by the factor 90°/α.

#### SNR assessment in phantoms

To measure the spatial distribution of SNR at increasing distances from the ^*19*^ *F*-*CRP* surface, a high-concentration ^19^F phantom (*Setup 1*) and an axial 2D-RARE scan (TR = 10 s, TE = 6.2 ms, ETL = 256, FOV = (25.6 × 25.6) mm^2^, matrix = 256 × 256, averages = 100, TA = 17 m) was used. To quantify and compare SNR in a way more relevant for brain inflammation, we measured SNR as a function of the number of ^19^F atoms using phantoms containing different concentrations of ^19^F nanoparticles (*Setup 2*, Fig. 1B). Measurements involved 2D-RARE scans (TR = 3000 ms, TE = 10.8 ms, ETL = 8, FOV = (10 × 10)mm^2^, matrix = 96 × 96, averages = 1, TA = 36 s) with varying slice thicknesses: 0.4/1.0/1.2/2.0/3.6/4.7/6.0 mm to measure SNR as a function of the number of ^19^F atoms.

SNR was calculated by dividing signal *S*_m_ from magnitude images by background standard deviation *σ*_m_, and corrected to compensate for the non-Gaussian distribution^29^. For single channel RF coils, intensity values of MR images follow a Rician distribution^30, 31^. For a two-receiver, quadrature system (^*19*^ *F*-*CRP*), they follow a non-central chi distribution^32^. We estimated the true SNR from the *S*_m_ and background *σ*_m_ using

2 $$SNR=\frac{S}{\sigma }=\frac{{S}_{{\rm{m}}}}{{\sigma }_{{\rm{m}}}}\cdot \frac{{f}_{{\rm{S}}}({S}_{{\rm{m}}},{\sigma }_{{\rm{m}}})}{1/{c}_{{\rm{\sigma }}}}$$

where *c*_*σ*_ is 0.655 (Rician) and 0.687 (chi), and the correction function *f*_S_ is derived from the respective distribution’s mean^30, 32^. For *Setup* 2, a single SNR value was determined from the mean signal intensity over a central circular region-of-interest covering ~90% of pixels. The number of atoms per image pixel was estimated from nanoparticle concentration and voxel size.

#### *Ex vivo* mouse brain ^19^F and ^1^H MRI (Setup 3)

^19^F MR images of the EAE mouse brain were acquired using 3D-RARE: TR = 800 ms, TE = 5.1 ms, ETL=33, FOV=(30 × 20 × 20) mm^3^, matrix = 195 × 65 × 65 zero-filled to 195 × 130 × 130, averages = 384, TA = 11 h. ^1^H MR images were acquired using 3D-FLASH (TR = 50 ms, TE = 12.5 ms, FOV = (30 × 20 × 20) mm^3^, matrix = 384 × 256 × 284 zero-filled to 768 × 512 × 512, averages = 2, TA = 6 h 3 min). ^19^F MR images from the ^*19*^ *F*-*CRP* were registered with those from the ^*19*^ *F*/^*1*^ *H RT*-*coil*. Since the ^*19*^ *F*-*CRP* has no ^*19*^ *F*/^*1*^ *H* dual resonant capacity, we registered the CRP ^19^F images onto the RT ^19^F images in order for both ^19^F images (RT and CRP) to be spatially aligned with the RT ^1^H images. For this, three repetitions of the RT ^19^F scan were averaged to achieve sufficient ^19^F signal with the RT-coil and an effective registration. Co-registration was applied using affine diffeomorphic image registration (12 degrees of freedom) by explicit B-spline regularization^33^, which is part of the Advanced Normalisation Tool (ANTs)^34^. Registration of the Allen brain atlas^35^ to the ^1^H image was achieved as follows: (1) ^1^H image and atlas template were segmented in grey matter (GM), white matter (WM) and cerebrospinal fluid (CSF) probability maps with SPMMouse (http://www.spmmouse.org/)^36^, (2) two synthetic images were generated with signal intensity in each voxel I(x,y,z) = 1.0 × GM (x y, z) + 2.0 × WM + 4.0 × CSF, i.e. one registered with the ^1^H image and one registered with the atlas, (3) both synthetic images were warped to the ^1^H image using nonlinear B-spline registration in ELASTIX (http://elastix.isi.uu.nl/)^37^. Raw ^1^H MRI files were converted to NIFTI-format and brains segmented with ITK-SNAP version 3.4.0^38^. For 2D representation of ^19^F/^1^H MRI we performed overlays of the raw ^19^F MR data with SNR-based scaling using Matlab. For 3D representation we used ImageJ (National Institutes of Health, USA, http://imagej.nih.gov/ij).

## Results

### Transmit field characteristics of the ^*19*^*F-CRP*

**Figure 2:**
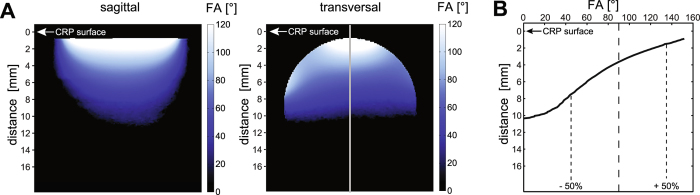
Transmission *B*_1_^+^ Field (*B*_1_^+^) for the ^*19*^ *F-CRP*. (**A**) Flip angle maps acquired in vertical and transversal orientation using a high concentration ^19^F phantom (Setup 1). (**B**) Profile plot of the FA along the vertical axis that depicts the change in FA with increasing distance to the CRP surface.

Since transceive surface coils do not achieve a spatially uniform excitation like volume resonators^24^, we assessed the *B*_1_^+^ characteristics of the ^*19*^ *F*-*CRP* (Fig. 2A) and quantified changes in FA. A profile plot of the FA along the vertical axis (Fig. 2B) reveals a strong FA decrease with increasing distance from the CRP surface. Across a distance of 10.4 mm the measured FA varies between 152° and 0°. From the nominal FA of 90° the actual FA deviates up to 50% within a range of 6.0 mm (1.5–7.5 mm from CRP surface).

### SNR assessment in phantoms

**Figure 3:**
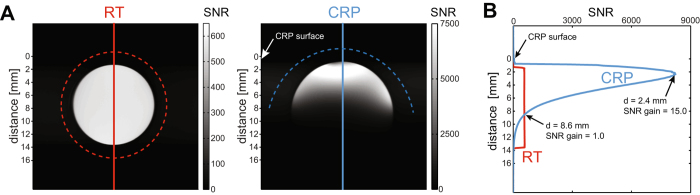
Comparison of SNR between the ^*19*^ *F-CRP* and ^*19*^ *F*/^*1*^ *H RT*-*coil*. (**A**) Cross-sectional spin-echo ^19^F MR images of a TFE phantom acquired with the RT RF coil (left) and the CRP (right). The CRP showed a spatially varying sensitivity that is typical for transceive surface coils. (**B**) Plots of the SNR profile along the vertical axis at the center of the phantom. For the RT volume resonator (red curve) the SNR was very uniform within the phantom. In contrast, for the CRP the SNR drops rapidly with increasing distance to the RF coil. For this particular reference pulse power, SNR reached its maximum at 2.4 mm from the CRP surface, where it is 15-fold higher than the SNR of the RT coil. Beyond a distance of 8.1 mm the ^*19*^ *F*-*CRP* did not provide any SNR gain with regard to the ^*19*^ *F*/^*1*^ *H RT*-*coil*.

To study the SNR performance of the ^*19*^ *F*-*CRP*, we first used a high ^19^F concentration (33% TFE solution) (Fig. 3A). The transversal spin-echo ^19^F MR images demonstrate a homogenous SNR for the RT coil and a spatially varying SNR for the CRP (Fig. 3A). We adjusted the reference pulse power in order to avoid substantial signal loss at the dorsal side of the brain. Using this reference pulse power, the SNR reached its peak at a distance of 2.4 mm, where it was ~15-fold higher than the SNR of the RT coil (Fig. 3B). The SNR of both RF coils are approximately equal at a distance of 8.6 mm from the CRP.

**Figure 4:**
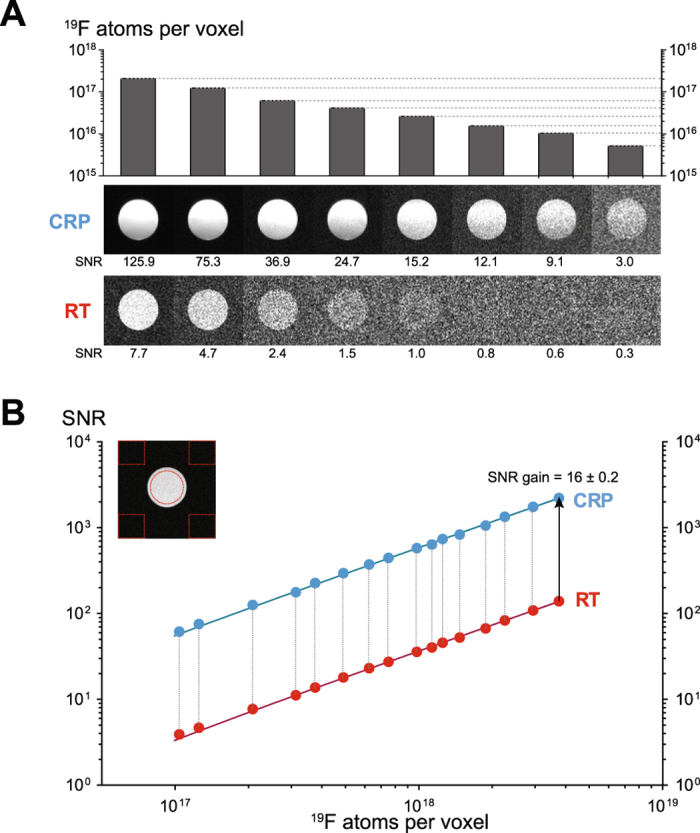
Comparison of ^19^F signal sensitivity between ^*19*^ *F-CRP* and ^*19*^ *F*/^*1*^ *H RT*-*coil* as a function of the number of ^19^F atoms. (**A**) Cross-sectional spin-echo ^19^F MR images of ^19^F nanoparticle phantoms acquired for both CRP (middle panel) and RT coil (lower panel). Each ^19^F MR image indicates an MR scan with a defined number of ^19^F atoms per voxel (upper panel) achieved with different concentrations of PFCE (ranging from 25 mM to 200 mM) and slice thicknesses varying from 0.4 to 2.0 mm. (**B**) Estimation of SNR gain provided by the ^*19*^ *F-CRP* compared to the ^*19*^ *F*/^*1*^ *H RT*-*coil* using high PFCE concentrations (200 mM to 1200 mM) and slice thicknesses varying from 1.0 to 6.0 mm. Shown is a log-log plot of SNR versus ^19^F atoms per voxel including a linear fit for both CRP (y = 5e^−16^x) and RT coil (y = 4e^−17^x).

We next investigated the detection limits for both coils by measuring ^19^F nanoparticles, as a biologically relevant preparation. We employed concentrations of PFCE (25 mM–200 mM) yielding a range of 10^15^–10^18 19^F atoms per voxel (Fig. 4A). Qualitatively, we reached a detection limit in the order of 10^15^ fluorine atoms using the ^*19*^ *F*-*CRP*, compared to 10^16^ fluorine atoms with the ^*19*^ *F RT*-*coil*. Specifically, an SNR of 3.0 was achieved with (0.1 × 0.1 × 0.4) mm³ voxels of a 25 mM PFCE concentration (equating to 5.2 × 10^15^ fluorine atoms) when using the ^*19*^ *F*-*CRP*. In contrast an SNR of 2.4 was achieved with (0.1 × 0.1 × 1.2) mm³ voxels of a 100 mM PFCE concentration (equating to 6.2 × 10^16^ fluorine atoms) when using the ^*19*^ *F*- *RT*-*coil*. In both cases the measurement time was 36 s. MR images with an SNR value below 2 were not sharply defined. To estimate SNR provided by the ^*19*^ *F*-*CRP* compared to the ^*19*^ *F*/^*1*^ *H RT*-*coil*, we used SNR = 2 as a cutoff equating to ~5 × 10^16^ (RT) and ~4 × 10^15^ (CRP) fluorine atoms per voxel. Next we prepared higher concentrations of ^19^F nanoparticles (200 mM to 1200 mM) to achieve SNR values well above 2, spanning a range of 10^17^–10^19^ atoms per voxel. From these experiments we calculated an SNR gain of ~16 for the ^*19*^ *F*-*CRP* when compared to the ^*19*^ *F*/^*1*^ *H RT*-*coil* (Fig. 4B).

### High spatially-resolved ^19^F MRI

**Figure 5:**
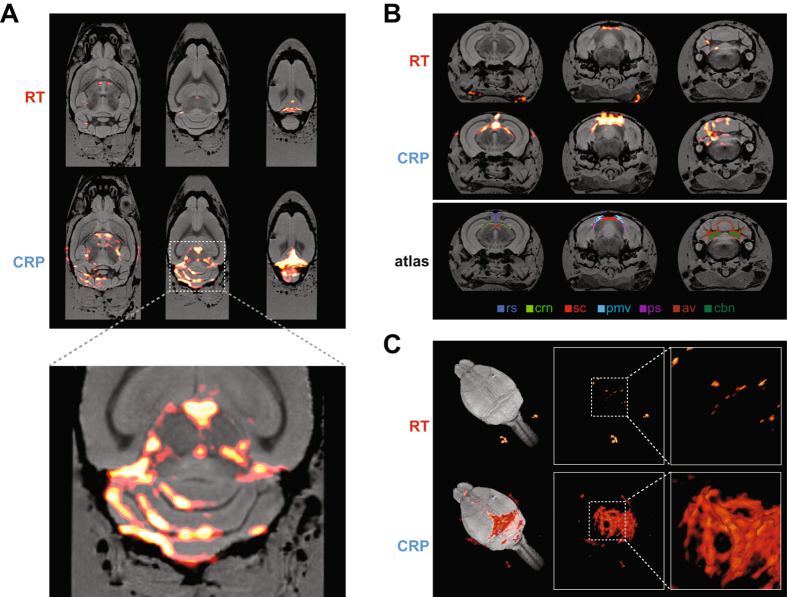
High spatial resolution ^19^F MR image of an *ex vivo* brain from an EAE mouse showing clinical disease. With both ^*19*^ *F*-*CRP* and ^*19*^ *F*/^*1*^ *H RT*-*coil*, ^19^F MR images were acquired using a 3D-RARE sequence. ^19^F MR images (shown in red) were combined with ^1^H MR images (shown in grayscale). ^1^H MR images were acquired using a 3D-FLASH sequence and the ^*19*^ *F*/^*1*^ *H RT*-*coil*. (**A**) Three exemplary slices from horizontal views of combined ^19^F/^1^H MR images for both ^*19*^ *F*/^*1*^ *H RT*-*coil* (*upper panel*) and ^*19*^ *F*-*CRP* (*middle panel*), in the *lower panel* a 300% zoom of the ^19^F/^1^H MR images acquired with the CRP. (**B**) Three exemplary slices from coronal views of combined ^19^F/^1^H MR images for both RT coil (*upper panel*) and CRP (*middle panel*). Registration of the Allen brain atlas to the ^1^H image (*lower panel*) shows following labelled brain regions: rs: retrosplenial area; crn: cranial nerves; sc: superior colliculus (sensory related); pmv: posteromedial visual area; ps: postsubiculum; av: arbor vitae; cbn: cerebellar nuclei. (**C**) 3-D rendering of the combined ^19^F/^1^H MR images for both ^*19*^ *F*/^*1*^ *H RT*-*coil* (*upper panel*) and ^*19*^ *F*-*CRP* (*lower panel*).

**Figure 6:**
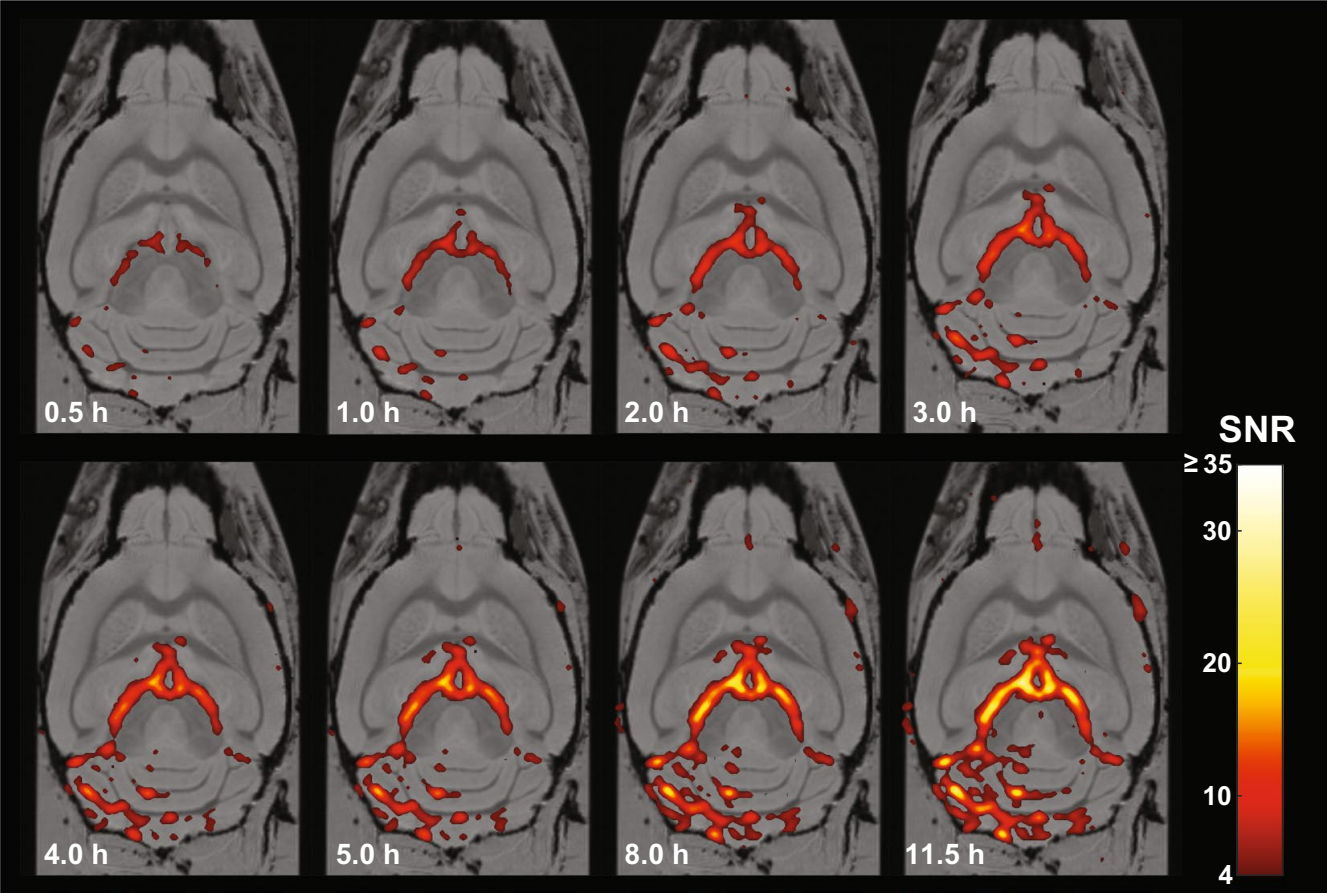
High spatial resolution ^19^F MRI using acquisition times feasible for *in vivo* imaging. ^19^F MR images were acquired with the ^*19*^ *F*-*CRP* using acquisition times between 30 min and 11 h. The ^19^F images were scaled to units of SNR, thresholded at SNR = 4, and overlayed onto the ^1^H MR images using a pseudocolor scale.

An important utilization of the SNR gain is to localize cell infiltrates in the brain with more detail. Previously areas of inflammation were detected using spatial resolutions greater than 600 μm^11^. Here we exploited the superior SNR of the ^*19*^ *F*-*CRP*, and used an isotropic spatial resolution of 150 μm. *Ex vivo* MR images obtained with the ^*19*^ *F*-*CRP* from an exemplary EAE mouse (day 10 following EAE induction, score = 1.25) show a more precise distribution of intraparenchymal inflammation. At this spatial resolution, the majority of the ^19^F signals obtained by the ^*19*^ *F*-*CRP* were not detected with the RT coil (Fig. 5A–C). In addition we show similar inflammatory patterns in a pre-symptomatic mouse, also sacrificed on day 10 following EAE induction (Supplementary Figure). Within the cerebellum, inflammatory infiltrates were mostly localized within the white matter of the arbor vitae, particularly near deep cerebellar nuclei (Fig. 5B). Clearly delineated inflammatory areas were found in grey matter regions running adjacent to white matter tracts in the cerebellum (Fig. 5A). This is consistent with the expected patterns of inflammation in the EAE model^39, 40^, also as observed in our own prior studies^11, 41, 42^. Using the ^*19*^ *F*-*CRP*, we also observed strong ^19^F signals in the cerebrum emanating from the striatum and pallidum appearing continuous with ^19^F signals from the third ventricle (Fig. 5A). Additionally, clear extraparenchymal meningeal inflammation could be seen, consistent with recent reports^43–45^. Especially strong inflammatory signals were observed along the dorsal surface of the brain, including meningeal regions lining fissures between the cerebellar lobules. These inflammatory regions extended ventrally to the prepyramidal fissure, parafloccular sulcus and lateral recess of the fourth ventricle. A dominant ^19^F signal was observed around the meninges lining the ventral part of the retrosplenial area of the cerebral cortex (Fig. 5B), spreading caudally towards the cerebellum, running in parallel to the superior sagittal sinus, and eventually the retroglenoid vein (Fig. 5C). In these experiments we focused on highly resolved inflammation imaging in the EAE brain, employing long acquisition times in order to compensate for the considerably lower ^19^F signal sensitivity of the ^*19*^ *F*/^*1*^ *H RT*-*coil*. Since these acquisition times (11 h) are not applicable for *in vivo* studies, we performed further experiments in which we reduced the scan time. Upon reducing the scan time from 11 h to 0.5 h we could still detect ^19^F signals with the ^*19*^ *F*-*CRP* (Fig. 6). Despite the clear differences we were nevertheless still able to detect a considerable ^19^F signal, even with a scan of only 2 h, which is amenable for *in vivo* MRI.

## Discussion

In this study we show first ^19^F MR images obtained with a ^*19*^ *F*-*CRP* driven in quadrature mode. Compared to the ^*19*^ *F*/^*1*^ *H RT*-*coil* we previously developed^11^, we show that the ^*19*^ *F*-*CRP* facilitates superior *ex vivo* images of brain inflammation in an animal model of MS. At the current stage of development the ^*19*^ *F*-*CRP* cannot yet be employed for *in vivo* imaging due to incompatibilities with conventional ^1^H RT coils, as discussed later. Nevertheless the results are encouraging, and offer proof-of-concept demonstration of the potential for this technology.

After introducing the concept of cryogenically-cooled RF coil hardware to reduce thermal noise and thus increase SNR^46^, CRP technologies were developed for small animal MRI, particularly for anatomical ^1^H MRI of mouse brain^41, 47–50^. Introducing a quadrature CRP design, enabled further SNR gains (~2.5) at 400 MHz^24, 25^ compared to RT coils with similar geometries. The SNR gain prediction for the ^*19*^ *F*-*CRP* is expected to be equivalent due to the close Larmor frequency (376 MHz at 9.4T).

The potential applications of ^19^F MR methods to image inflammation have long been recognized^11–17^. For several years, neuroinflammation has been studied using gadolinium-based contrast agents. However, gadolinium-enhancing lesions are diffuse, and lack spatial precision. Improvements have been realized with the use of alternative contrast agents, such iron oxide nanoparticles, although their effects on magnetic susceptibility limit their discrimination from endogenous confounding artifacts. ^19^F MR methods abrogate this, since ^19^F signals derive exclusively from exogenously applied ^19^F nanoparticles. Efforts have been made to boost ^19^F signal e.g. by promoting ^19^F nanoparticle cellular uptake^20^. Nevertheless, major challenges of signal sensitivity constraints remain. Improving ^19^F sensitivity with the ^*19*^ *F*-*CRP* will be essential to realizing the full potential of ^19^F MR.

Our motivation to investigate the ^*19*^ *F*-*CRP* was to increase the sensitivity to detect neuroinflammation. Considering the geometrical differences between both coils, it was imperative to measure SNR at locations below the CRP that correspond to the mouse brain, using phantoms spanning the entire coronal view, as a basis for future *in vivo* studies. We performed SNR measurements for both ^*19*^ *F*-*CRP* and control ^*19*^ *F*/^*1*^ *H RT*-*coil* using a spin echo sequence (RARE), commonly used for ^19^F MRI due to its high SNR per unit time compared to spoiled gradient echo sequences.

The sensitivity of the ^*19*^ *F*-*CRP* is spatially dependent. Given that the CRP is a transceive quadrature surface coil array, both transmit field (*B*_1_^+^) and receive sensitivity (*B*_1_^−^) diminish with increasing distance from the RF coil – a factor that must be accounted for in quantitative imaging by measuring the actual *B*_1_ and correcting the signal intensities using the signal equation of the employed pulse sequence. This is absolutely essential when signal quantification is necessary in order to ascertain the level of inflammation over the entire region of the brain during EAE. Nevertheless, this characteristic is shared by all transceive surface coils. This adverse effect is counterbalanced by an SNR gain, up to ~15-fold in the practical comparison made within this study. This SNR gain can be attributed to factors including cooling (in the range of 2–3 for ^1^H^24, 25^), differences in RF coil design (birdcage vs. surface coil; quadrature versus linear), RF coil sample loading, and the specific RF pulse power adjustments. Here, pulse power was adjusted in order to avoid substantial signal loss at the dorsal part of the brain, which is observed when using a RARE sequence with excessive RF power. Predicting the sensitivity and detection limits of ^19^F measurements for specific hardware setups^51^ will help facilitate further ^*19*^ *F*-*CRP* studies with other fluorinated compounds.

An SNR gain of 15 can be exploited in several ways — by reducing scan time by a factor 225 (e.g. from 1 h to ~15 s), or doubling 3D spatial resolution (e.g. from 600 µm to 300 µm) while still gaining SNR (~2.5). In this study we made use of the superior SNR, employing isotropic spatial resolutions of 150 μm to study neuroinflammation. Using the ^*19*^ *F*-*CRP* at this resolution, we gained more precise information regarding inflammatory cell localization in the brain, compared to our previous study^11^. The ^19^F MR images with the CRP showed excellent correspondence with the typical pattern of histopathology^39, 40^. A robust accumulation of inflammatory lesions, especially in the white matter tracts of the cerebellum, is a hallmark of EAE in SJL mice, which we also observed in our prior studies using high resolution ^1^H MR^41, 42^ and low resolution ^19^F MR^11^. The pathology also extends into the cerebrum, as shown both prior to the occurrence of clinical symptoms (Supplementary Figure) and also during ongoing clinical disease (Figs 5 and 6). The ^*19*^ *F*-*CRP* MR images also enabled discrimination of extraparenchymal meningeal inflammation, consistent with recent reports highlighting the relevance of inflammatory cell trafficking via the blood meningeal barrier^43, 44^ and extravasation via leptomeningeal microvessels into the subarachnoid space^45^. This also reflects the situation in MS^3–5^. Recent studies have argued for the presence of a lymphatic circulation in the meninges in association with these vessels, capable of draining immune cells from meningeal spaces^8^ and brain parenchyma^7^ into deep cervical lymph nodes. Therefore, the capacity to perform non-invasive longitudinal investigations with fidelity ^19^F MRI to monitor the dynamics and distribution of infiltrating immune cells will be directly relevant for experimental neuroimmunologists.

The gradient in the *B*_1_ field of the ^*19*^ *F*-*CRP* leads to a gradual decline in ^19^F MR signal with increasing distance from the probe head. This results in reduced signal in ventral regions. Studies of the EAE model are, in general, more focused on imaging of the CNS, and less so on imaging of the superficial lymph nodes. When imaging of the lymph nodes in the ventral regions is necessary, one could consider measuring the mouse brain in the supine and prone positions, in order to ensure coverage of the dorsal sides comprising the whole brain as well as ventral sides to include the draining lymph nodes. Other possible workarounds include adding an anterior ^19^F RT RF coil to the mouse bed or combining ^19^F images from RT and CRP. These approaches could help to overcome this inherent limitation of the ^*19*^ *F*-*CRP*, while still utilizing its superior SNR. While the spatial dependency poses a constraint for studies investigating the involvement of the draining lymph nodes, the translational applications of the ^*19*^ *F*-*CRP* are not limited to EAE. The ^*19*^ *F*-*CRP* will also be useful for studying brain inflammation in animal models of tumour growth (especially those tumours implanted in the cortex or striatum), and studies on the middle cerebral artery occlusion model of stroke. Inflammation in these preclinical models could readily be imaged, since the focus of pathology in these models is located in regions where the ^*19*^ *F*-*CRP* clearly outperforms the ^*19*^ *F*/^*1*^ *H RT*-*coil*.

*In vivo* ^19^F MRI studies require acquisition of anatomical ^1^H MR images within a reasonable time frame. A dual-tunable RF probe would be most ideal, in order to avoid inaccurate co-registration of both signals^52^. Despite the clear improvement in SNR of the ^*19*^ *F*-*CRP*, the quadrature design prohibits the presence of a dual resonant MR signal that would be needed for anatomical ^1^H MRI. Furthermore conventional ^1^H RF resonators cannot be used in combination with the ^*19*^ *F*-*CRP* due to coupling between both RF coils. To avoid this, the ^*19*^ *F*-*CRP* would need to be removed while the *in vivo* ^1^H images are acquired. This would cause changes in the alignment of the mouse within the scanner during *in vivo* measurements that are serious enough to constitute a major hindrance. Even with the use of reference markers, any slight shift in the position of the markers with respect to the mouse during the procedure will result in an incorrect registration between ^19^F and ^1^H images. The current procedure of registering the ^19^F images of the CRP with those of the RT RF coil is complicated and time consuming, requires sufficient SNR and is an impediment for *in vivo* experiments. A proposed solution to this limitation could be to construct an anterior ^1^H RT RF coil, specifically designed to be added to the mouse bed while the ^*19*^ *F*-*CRP* remains installed, in order to provide anatomical guidance. A dual-tunable ^1^H/^19^F RT RF coil would also take into account the above approach (implementation of a ^19^F RF-coil below the mouse head).

This study presents the first demonstration of the performance of a quadrature ^*19*^ *F*-*CRP* tailored for small rodents, showing superior SNR and ^19^F MR image quality. The logical extension of this work will be to translate these results into *in vivo* studies, such as those studying pathological changes during neuroinflammatory disease. While the results of the current study are highly encouraging, a challenging road still lies ahead for the application of the ^*19*^ *F*-*CRP* in *in vivo* studies. Previous studies using ^19^F MR have been seriously hampered by the low SNR, and compensating for this limitation by using low spatial resolution has generally yielded images with rather poor definition, and therefore limited scientific utility. The current study aims to improve this situation, bringing ^19^F MR imaging a step closer to the objective of ‘microscopic MRI’. Our results showed a remarkable SNR and detail of neuroinflammation, compared to conventional ^19^F MRI, heralding a bright potential for the application of ^*19*^ *F*-*CRP* for non-invasive MRI *in vivo*.
